# Circulating miR-185 might be a novel biomarker for clinical outcome in patients with dilated cardiomyopathy

**DOI:** 10.1038/srep33580

**Published:** 2016-09-20

**Authors:** Miao Yu, Wei Liang, Yu Xie, Qi Long, Xiang Cheng, Yu-Hua Liao, Jing Yuan

**Affiliations:** 1 Laboratory of Cardiovascular Immunology, Institute of Cardiology, Union Hospital, Tongji Medical College, Huazhong University of Science and Technology, Wuhan, 430022, China

## Abstract

B cells contribute to the development of dilated cardiomyopathy (DCM) by inducing myocyte injuries and myocardial fibrosis. Our recent research indicated that microRNA (miR) -185 participated in human B-cell activation. Thus, this study was aimed to explore the relationship between miR-185 and DCM progression. Forty-one healthy volunteers and fifty newly diagnosed DCM patients were enrolled. The levels of plasma miR-185, TNF-α secreting B cells, and anti-heart autoantibody were detected. We found that the mean levels of plasma miR-185 in DCM patients were significantly higher than those in healthy controls. Furthermore, these DCM patients could be divided into miR-185^high^ and miR-185^low^ groups according to the cluster distribution. During one-year follow-up period, the miR-185^high^ group showed apparent improvements in left ventricular ejection fraction, left ventricular end diastolic diameter, and NT-proBNP, accompanied by significant declines in both cardiovascular mortality and total admissions for heart failure re-hospitalizations. In addition, the levels of anti-β1-AR antibody and TNF-α secreting B cells were also reduced in miR-185^high^ group. These findings suggested that high miR-185 levels might be associated with a favorable prognosis by repressing B cell function in DCM. The findings of this study need to be confirmed with larger sample size and longer duration of observation.

Dilated cardiomyopathy (DCM), a primary cause of heart failure, is characterized by left ventricular dilation and systolic dysfunction^1^. Nowadays, despite a great deal of progress in the treatment of heart failure, the prognosis of most DCM patients remains poor. Immune inflammation mediated by T cells plays an important role in the development of heart failure. However, in DCM, B cells also contribute to myocyte injuries and myocardial fibrosis by generating anti-heart autoantibodies (AHA) and secreting TNF-α^2^,^3^,^4^.

MicroRNA (miR) is a non-coding RNA regulating target gene expression post-transcriptionally^5^. Due to their stability and detectability, several immune regulatory miRs in peripheral blood have shown a potential role in cardiac remodeling. Miyamoto *et al.* recently reported that circulating miR-155, miR-639, miR-636, and miR-646 could be useful biomarkers for recovery in pediatric DCM^6^. Satoh *et al.* even revealed that a decrease in let-7i implied poor clinical outcomes in DCM patients^7^. But today the functions of microRNAs in DCM are still not clear.

MiR-185, located in the 22q11.2 gene locus^8^, is generally regarded as a regulator involved in the biological processes of carcinoma cells and neurological disorders^9^,^10^. Recently, we screened and found that miR-185 participated in human B-cell activation by targeting EphB2 in B cells^11^. This finding suggested that miR-185 might have an influence on B cell function and the pathogenic process of DCM. So in this study, we enrolled DCM patients, detected the circulating miR-185 expressions during the course of disease, and explored the relationship between miR-185 and DCM progression.

## Methods

### Patients

Fifty newly diagnosed patients with DCM in Union Hospital, Tongji Medical College, Huazhong University of Science and Technology from February 2013 to February 2014 were recruited for this study. Each of them was diagnosed with DCM based on the 1995 WHO/ISFC criteria^12^. Patients with any other acute or chronic diseases such as tumors, infection, hematopathy, rheumatic diseases, etc. were excluded. None of the patients had been treated with immunosuppressors or anti-inflammatory drugs. All patients were treated with the standard therapy including angiotensin-converting-enzyme inhibitors/angiotensin receptor blockers (ACEI/ARBs), β-blockers, and aldosterone antagonist. If necessary, diuretics, digitalis, and nitrates were administered for attenuating the symptoms of heart failure. Forty-one age and gender-matched healthy volunteers were enrolled as the controls. All the participants underwent a follow-up examination at 3, 6, and 12 months. The study was conducted in accordance with the guidelines of the Helsinki declaration and its amendments. The Ethics Committee of Tongji Medical College of Huazhong University of Science and Technology approved this research, and the informed consent was obtained from each subject.

### Blood Samples

Blood samples were obtained from all of the recruited DCM patients and healthy volunteers, in a fasting state, in the morning at the hospital with a 21-gauge needle for clean antecubital venipuncture in a vial containing 3.2% sodium citrate. The same procedure was followed for blood draws in the follow-up period. Each sample consisting of 4 ml blood was collected and centrifuged at 2000 rpm for 15 min, and the isolated plasma was stored at −20 °C for further measurements, and cells that remained were layered over Ficoll–Hypaque density gradient solution to separate peripheral blood mononuclear cells (PBMCs).

### B cells isolation

The PBMCs from DCM patients were prepared for B cell purification by using the human naïve B cells isolation kit (Miltenyi Biotech, Germany), according to the manufacturer’s protocol. Briefly, PBMCs were incubated with B cell biotin-antibody cocktail for 15 minutes and followed by anti-biotin microbeads for 10 minutes at 4 °C. After that, these cells were resuspended in MACS buffer and loaded on a LS column (Miltenyi, Germany) for purification. The purity of the isolated B cell population was over 95%.

### RNA isolation and TaqMan microRNA assays

Total RNA from plasma and B cells of the blood samples was respectively extracted with TRIzol reagent (Invitrogen), according to the manufacturer’s instructions. The purity and concentration of the RNA samples were measured by UV spectrophotometer, and a ratio of A260/280 ranging from 1.8 to 2.0 was considered acceptable. The reverse transcription was performed using the TaqMan MicroRNA Reverse Transcription kit components, following the manufacturer’s protocol. The reverse transcription system was incubated at 16 °C for 30 min, 42 °C for 30 min, and 85 °C for 5 min. The absolutely quantitative PCR (qPCR) was carried out using the TaqMan Universal PCR Master Mix Kit (Applied Biosystems, miR-185 primer Cat#4427975, ID: 002271), and 20μl of the dilutional linearities to miR-185 standard (Integrated Biotech Solutions Co, Shanghai, China) or test samples were run on a 7300HT analyzer (Applied Biosystems, CA, USA). The levels of miR-185 were determined using standard curves.

### AlphaLISA

The plasma levels of TNF-α were measured by customized AlphaLISA assay kits (PerkinElmer BioSignal Inc, USA), following the manufacturer’s instructions. The lower detection limit (LDL) was 2.2 pg/ml. The resulting plates were read using the AlphaLISA detection mode on Envision plate reader (PerkinElmer BioSignal Inc, USA).

### Flow Cytometry

The PBMCs from DCM patients were harvested and stained with FITC-labeled anti-human CD19 antibody (eBioscience, CA, USA). After washing, the cells were resuspended at a density of 2 × 10^6^/ml and stimulated with 100 ng/ml PMA, 1 μg/ml ionomycin and 1 μg/ml monensin (all from eBioscience, CA, USA) at 37 °C, 5% CO2 of a 24-well culture plate (Corning) in RPMI 1640 medium (Gibco) supplemented with 100 U/ml penicillin, 100 μg/ml streptomycin, and 10% FCS (Gibco). After 5 h, the cells were fixed, permeabilized and stained intracellularly with PE-Cy7 labeled anti-human TNF-α antibodies (eBioscience, CA, USA) at 4 °C for 30 min. Meanwhile, the isotype control antibodies (eBioscience) were used to ensure the specificity of the staining. Finally, the cells were washed and analyzed by FACScalibur flow cytometry.

### ELISA

ELISA method was used to examine the levels of plasma anti-heart antibody (AHA) including antibodies (Abs) against the adenine nucleotide translocator (ANT), β1 adrenergic receptor (β1-AR), myosin heavy chain (MHC) and L-type calcium channel (CC) according to the procedures described in previous studies^13^,^14^,^15^,^16^,^17^. Briefly, the peptides derived from different human cardiac proteins including ANT (PIERVKLLLQ-YDEIKKFV), β1-AR (HWWRAESDEARRCYNDPKCCDFVTN RC), MHC (EIERKLAEKD-VDKLQLKV-AKSRDIGAKGLNE) and CC (VNENTRMYIPEENHQ) were synthesized by a PSSM-8 automated peptide synthesizer (Shimadzu, Japan). The purities of the synthetic peptides confirmed by high liquid chromatography were up to 96%. A horseradish peroxidase-labeled rat antihuman IgG (Gibco, USA) was used to detect AHA, and the absorbance (A) of the dye is measured at a wavelength of 450 nm by using a Beckman DU-600 Spectrophotometer. All of the samples were measured in triplicate.

### Statistical analysis

SPSS11.0 was used for all statistical analyses. All data were presented as mean ± SEM. Chi-square tests were performed for the categoric variables of clinical characteristics including sex, virology and re-hospitalization. Bivariate correlation analysis was used as a test of correlation between two variables. Mann-Whitney rank sum test was performed for NYHA classification. Survival analyses were made by using the Kaplan-Meier method. 2-tailed Student’s t test was performed for the mean comparison in two groups, and one-way ANOVA was performed for the mean comparison in three groups. Differences were considered significant at a value of P < 0.05.

## Results

### The clinical features of DCM patients and healthy volunteers

The clinical data of healthy volunteers and patients with DCM are listed in Table 1. Compared with healthy controls, DCM patients showed significant depressed left ventricular ejection fraction (LVEF, P < 0.001), increased left ventricular end-diastolic dimension (LVEDD, P < 0.001), elevated N-terminal of the prohormone brain natriuretic peptide (NT-proBNP, P < 0.001), enhanced CRP (C-reactive protein, P < 0.001) and boosted viral infection rates including coxsackie-B3 (P < 0.001), coxsackie-B5 (P = 0.001), cytomegalovirus (P = 0.002) and enteric virus (P < 0.001). There were no differences in age (P = 0.311) and sex (P = 0.920) between healthy controls and DCM patients.

### Circulating miR-185 expressions at baseline

**Figure 1:**
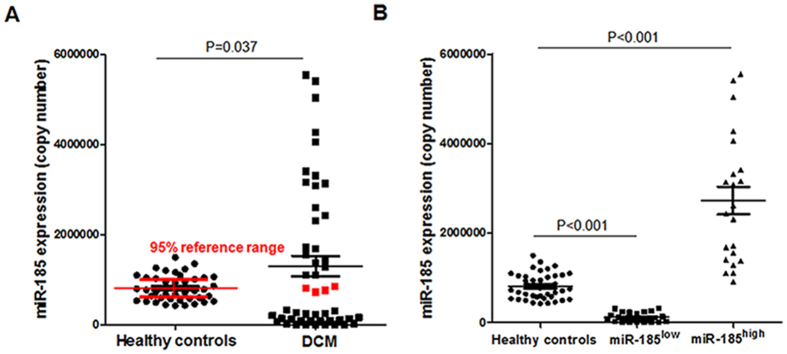
MicroRNA-185 Expression in Peripheral Blood of DCM Patients. (**A**) MicroRNA-185 expressions were investigated in peripheral blood of healthy controls and DCM patients by TaqMan^®^ microRNA assays at the time of enrollment (P = 0.037). (**B**) According to the levels of miR-185, DCM patients were divided into miR-185^high^ group and miR-185^low^ group (P < 0.001). The results are the mean ± SEM.

MiR-185 was detectable in the peripheral blood of both healthy controls and DCM patients, and the levels of miR-185 were significantly higher in DCM patients (P = 0.037, Fig. 1A). Interestingly, only four DCM patients whose miR-185 levels lay within the 95% reference range of healthy controls [721943, 897824] (copy number of miR-185). In a majority of DCM patients, the miR-185 levels were seen to be distributing into two clusters, respectively, either obviously higher or lower than those in healthy controls (P < 0.001, Fig. 1A,B).

### The relationship between miR-185 and cardiac function in DCM patients

**Figure 2:**
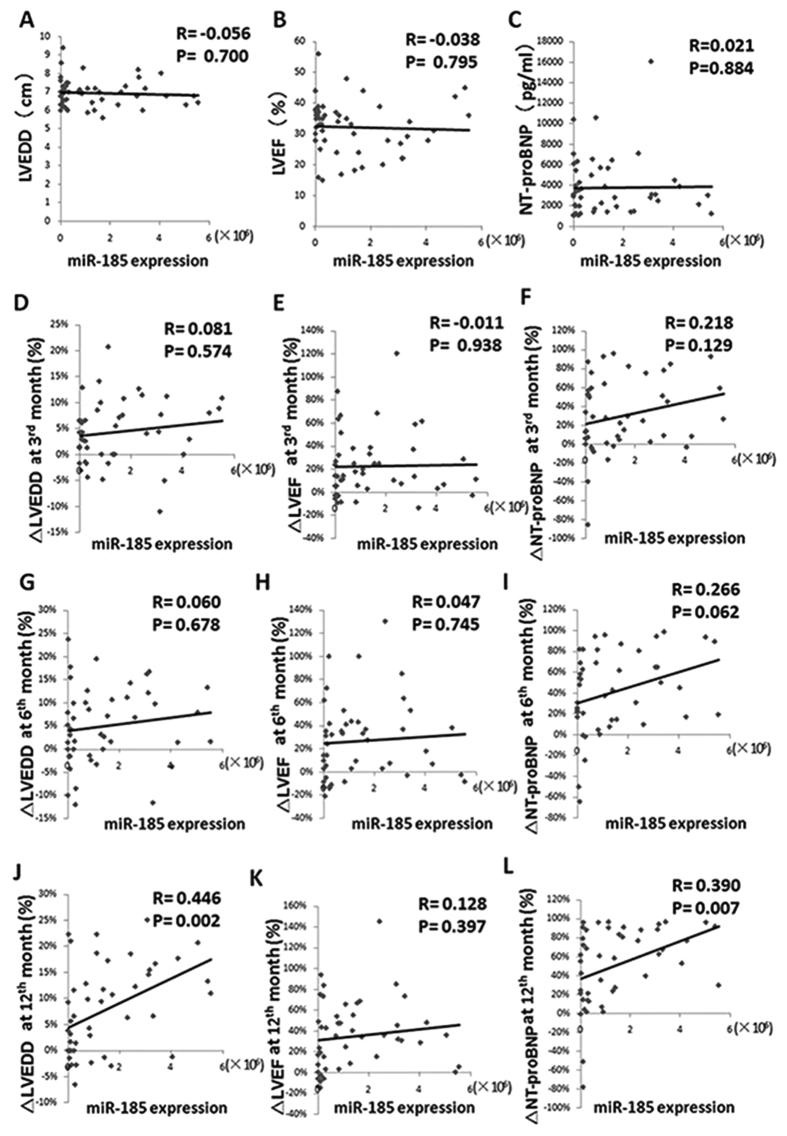
The Correlation of MiR-185 Levels and the Indexes of Cardiac Function in DCM Patients. (**A–C**) The correlation analysis of miR-185 levels and LVEDD, LVEF and NT-proBNP at month 0 in DCM patients, the correlation index (R) is respectively −0.056, −0.038 and 0.021, all P > 0.05. (**D–F**) The correlation analysis of miR-185 levels and the reduction rates of LVEDD (R = 0.081), LVEF (R = −0.011) and NT-proBNP (R = 0.218) at 3^rd^ month compared with the baselines at month 0 in DCM patients, all P > 0.05. (**G–I**) The correlation analysis of miR-185 levels and the reduction rates of LVEDD (R = 0.060), LVEF (R = 0.047) and NT-proBNP (R = 0.266) at 6^th^ month compared with the baselines at month 0 in DCM patients, all P > 0.05. (**J–L**) The correlation analysis of miR-185 levels and the reduction rates of LVEDD (R = 0.446, P = 0.002), LVEF (R = 0.128, P > 0.05) and NT-proBNP (R = 0.390, P = 0.007) at 12^th^ month compared with the baselines at month 0 in DCM patients. ΔLVEDD = (the baseline LVEDD at month 0- LVEDD in the follow-up period)/the baseline LVEDD at month 0, ΔLVEF = (LVEF in the follow-up period-the baseline LVEF at month 0)/the baseline LVEF at month 0, and ΔNT-proBNP = (the baseline NT-proBNP at month 0- NT-proBNP in the follow-up period)/the baseline NT-proBNP at month 0.

We performed correlation analysis of miR-185 levels and the indexes of cardiac function LVEDD, LVEF, and NT-proBNP in patients with DCM at baseline. However, the miR-185 levels were not correlated with the levels of these three indexes (Fig. 2A–C).

Subsequently, compared with the baselines at month 0, we calculated the alteration rates of LVEDD, LVEF and NT-proBNP at 3, 6, and 12 months. The baseline miR-185 levels were positively correlated with the reduction rate of LVEDD (R = 0.446, P = 0.002) and NT-proBNP (R = 0.390, P = 0.007) in DCM patients at 12 months. However, the baseline miR-185 levels were not correlated with alterations of LVEDD and NT-proBNP at 3 and 6 months. In addition, the correlations between miR-185 levels and LVEF were not significant in the follow-up period (Fig. 2D–L).

### The clinical features of miR-185^high^ and miR-185^low^ DCM patients

To further explore whether the higher miR-185 levels would lead to the better clinical outcomes in DCM, we classified these forty-six cases of DCM patients into miR-185^high^ group and miR-185^low^ group according to the miR-185 data distribution in Fig. 1. The clinical data of patients in miR-185^high^ and miR-185^low^ groups at baseline are listed in Table 2. There were no differences in the clinical features including age, sex, viral infection rates, NYHA classification, LVEF, LVEDD, NT-proBNP and medications between them at baseline. However, the levels of anti-β1-AR antibody (P = 0.022) and plasma CRP (P < 0.001) in miR-185^low^ group were significantly higher than those in miR-185^high^ group.

### Mortality in miR-185^high^ and miR-185^low^ DCM patients at 12-month follow-up

**Figure 3:**
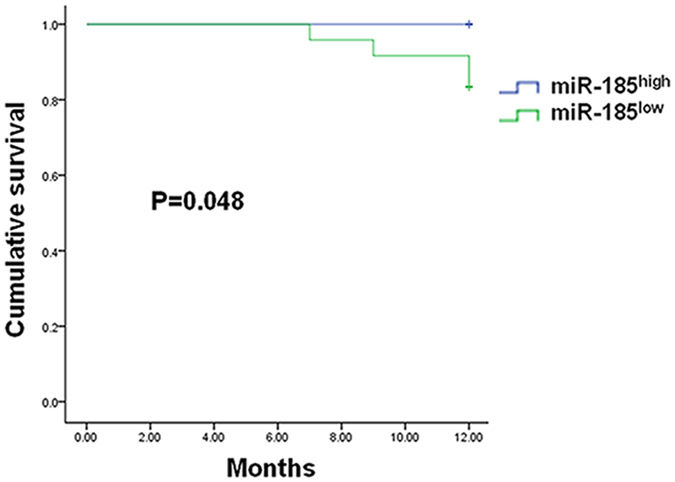
Kaplan-Meier Survival Curves for MiR-185high and MiR-185^low^ Patients.

During the one-year observation period, a total of four (16.7%) miR-185^low^ patients died while none of the miR-185^high^ patients died. All of the four miR-185^low^ patients died from cardiovascular causes (cardiogenic shock at the 7^th^ month, cardiogenic shock at the 9^th^ month, ventricular fibrillation at the 12^th^ month, and cardiac arrest at the 12^th^ month respectively). Figure 3 depicts Kaplan-Meier survival curves for the two groups (P = 0.048).

### Heart failure re-hospitalization in miR-185^high^ and miR-185^low^ DCM patients at 12-month follow-up

Among the 46 patients who completed the one-year follow-up, 6 (27.3%) miR-185^high^ patients and 10 (41.7%) miR-185^low^ patients were re-hospitalized because of heart failure at least once over the year, and some of them were re-hospitalized more than once during this period. Thus, among these total admissions for heart failure re-hospitalizations, there were 8 (36.4%) admissions in miR-185^high^ group, which was significantly lower than those of miR-185^low^ group with 17 (72.9%) admissions (P = 0.011, Table 3).

### Cardiac function in miR-185^high^ and miR-185^low^ DCM patients at 12-month follow-up

**Figure 4:**
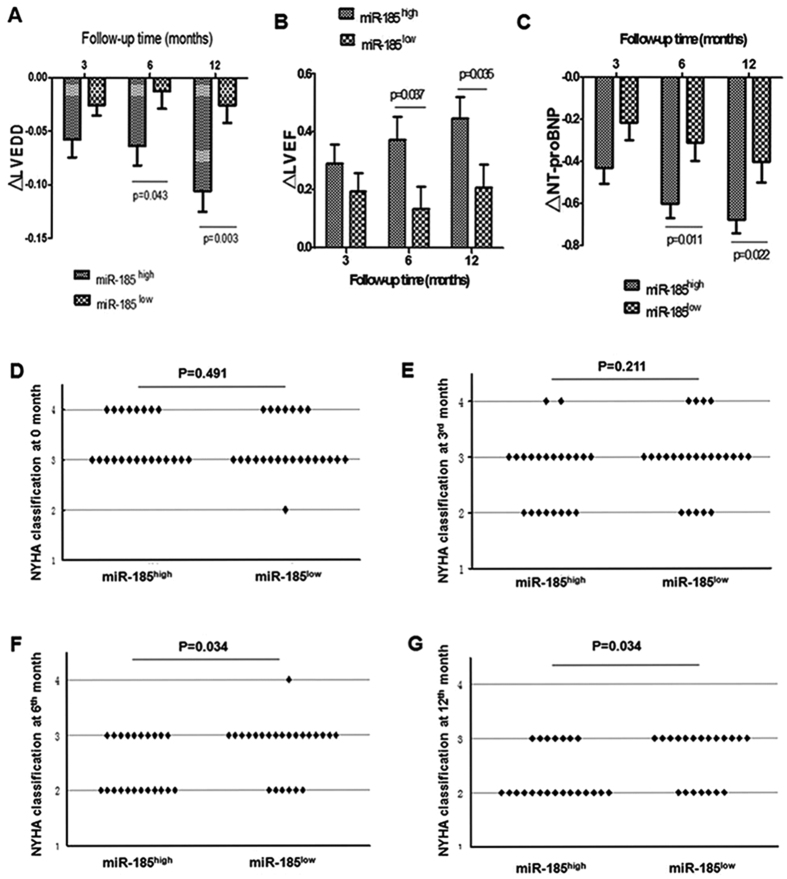
A Follow-up Study of Cardiac Function in MiR-185^high^ and MiR-185^low^ Patients. (**A**) The reduction of LVEDD compared to that at the time of enrollment in miR-185^high^ and miR-185^low^ groups at 3^rd^ (P > 0.05), 6^th^ (P = 0.043) and 12^th^ (P = 0.003) months. (**B**) The elevation of LVEF compared to that at the time of enrollment in miR-185^high^ and miR-185^low^ groups at 3^rd^ (P > 0.05), 6^th^ (P = 0.037) and 12^th^ (P = 0.035) months. (**C**) The reduction of NT-proBNP compared to that at the time of enrollment in miR-185^high^ and miR-185^low^ groups at 3^rd^ (P > 0.05), 6^th^ (P = 0.011) and 12^th^ (P = 0.022) months. (**D–G**) NYHA classification of the patients evaluated respectively at 0 (P > 0.05), 3^rd^ (P > 0.05), 6^th^ (P = 0.034) and 12^th^ (P = 0.034) months. ΔLVEDD = (LVEDD in the follow-up period-the baseline LVEDD at month 0)/the baseline LVEDD at month 0, ΔLVEF = (LVEF in the follow-up period-the baseline LVEF at month 0)/the baseline LVEF at month 0, and ΔNT-proBNP = (NT-proBNP in the follow-up period-the baseline NT-proBNP at month 0)/the baseline NT-proBNP at month 0. Values are means ± SEM.

In the one-year follow-up, we collected the indexes of cardiac function including LVEDD, LVEF, NT-proBNP and NYHA classification in patients with DCM at 0, 3, 6, and 12 months. Compared with the baselines at month 0, we calculated the change of LVEDD, LVEF, and NT-proBNP at 3, 6, and 12 months. A more significant reduction of LVEDD (P = 0.043 for the 6^th^ month, P = 0.003 for the 12^th^ month, Fig. 4A) and NT-proBNP (P = 0.011 for the 6^th^ month, P = 0.022 for the 12^th^ month, Fig. 4C), and more obvious elevations of LVEF (P = 0.037 for the 6^th^ month, P = 0.035 for the 12^th^ month, Fig. 4B) in miR-185^high^ group at the 6^th^ and 12^th^ months were observed. There were no differences in these four indexes between miR-185^high^ group and miR-185^low^ group at the 3^rd^ month compared to baselines at month 0. In addition, the numbers of miR-185^high^ patients were increased in NYHA classification II while decreased in NYHA classification III compared with those of miR-185^low^ patients at 6^th^ (P = 0.034) and 12^th^ months (P = 0.034), but no significant alteration was found at the 3^rd^ month (Fig. 4D–G).

### The relationship between miR-185 and B cell function

**Figure 5:**
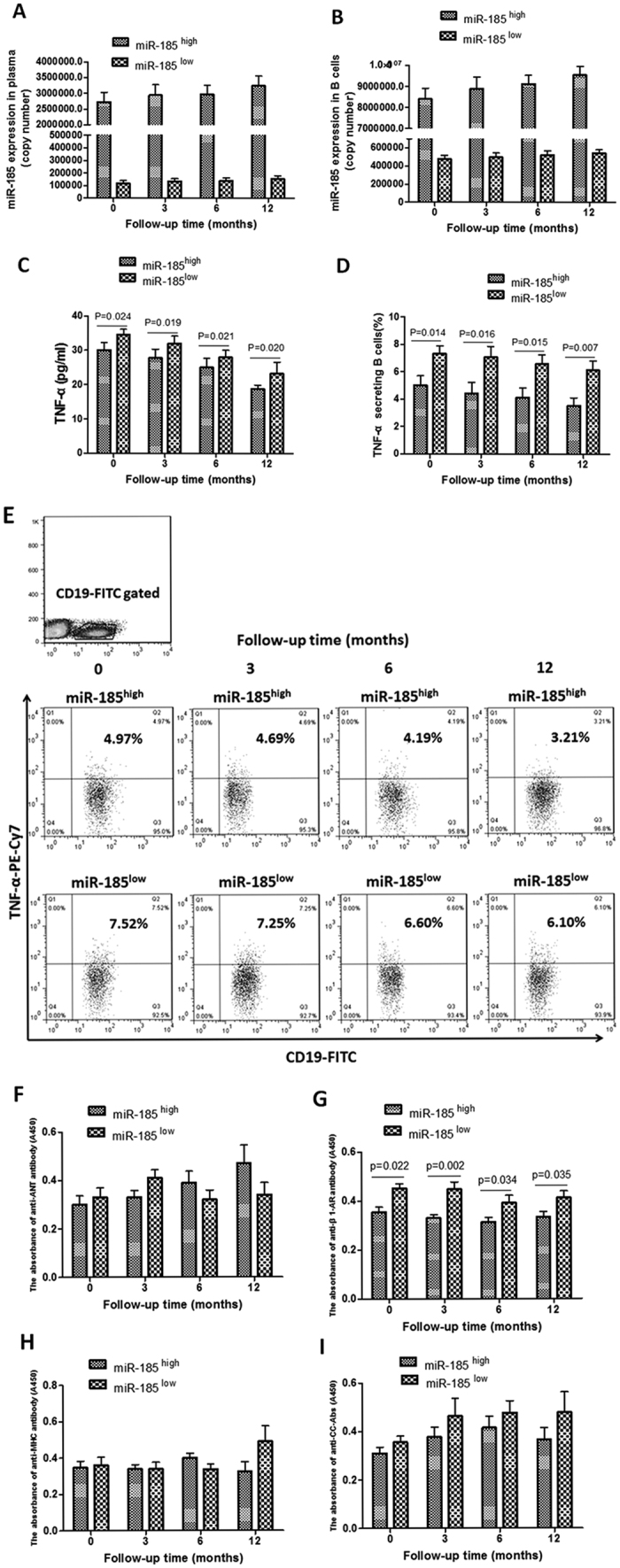
The Relationship between MiR-185 and B cell Function in DCM Patients. (**A**) The levels of miR-185 in plasma were not changed during the follow-up period (P > 0.05). (**B**) The levels of miR-185 in B cells were not changed during the follow-up period (P > 0.05). (**C**) The levels of plasma TNF-α in miR-185^high^ and miR-185^low^ groups respectively at 0 (P = 0.024), 3^rd^ (P = 0.019), 6^th^ (P = 0.021) and 12^th^ (P = 0.020) months. (**D**) The results of statistical analysis for the frequencies of TNF-α secreting B cells in miR-185^high^ and miR-185^low^ groups respectively at 0 (P = 0.014), 3^rd^ (P = 0.016), 6^th^ (P = 0.015) and 12^th^ (P = 0.007) months. (**E**) Representative pictures from DCM patients for the frequencies of B cells expressing TNF-α. (**F**) The levels of plasma anti-ANT antibody detected by ELISA respectively at 0, 3^rd^, 6^th^ and 12^th^ months, all P > 0.05. (**G**) The levels of plasma anti-β1-AR antibody detected by ELISA respectively at 0 (P = 0.022), 3^rd^ (P = 0.002), 6^th^ (P = 0.034) and 12^th^ (P = 0.035) months. (**H**) The levels of plasma MHC antibody detected by ELISA respectively at 0, 3^rd^, 6^th^ and 12^th^ months, all P > 0.05. (**I**) The levels of plasma anti-calcium channel antibody detected by ELISA respectively at 0, 3^rd^, 6^th^ and 12^th^ months, all P > 0.05. Values are means ± SEM.

There was no significant change in the miR-185 levels in plasma and B cells at the month 3, 6, and 12 compared to the baselines in the follow-up period (Fig. 5A,B). Compared with the miR-185^low^ group, the levels of plasma TNF-α (P = 0.024 for month 0, P = 0.019 for 3^rd^ month, P = 0.021 for 6^th^ month, and P = 0.020 for 12^th^ month) and TNF-α secreting B cells (P = 0.014 for month 0, P = 0.016 for 3^rd^ month, P = 0.015 for 6^th^ month, and P = 0.007 for 12^th^ month) were lower in the miR-185^high^ group at the 3^rd^, 6^th^ and 12^th^ months, respectively (Fig. 5C,D). However, no significant change was found in the levels of plasma TNF-α and TNF-α secreting B cells at the 3^rd^, 6^th^ and 12^th^ months as compared to the baselines during the follow-up period in both the miR-185^high^ and the miR-185^low^ groups (Fig. 5C–E).

Compared with the miR-185^low^ group, the levels of anti-β1-AR antibody were significantly lower at month 0 (P = 0.022), 3^rd^ (P = 0.002), 6^th^ (P = 0.034), and 12^th^ (P = 0.035) months in the miR-185^high^ group. However, no significant change was found in the anti-ANT, MHC and CC antibodies between the miR-185^low^ and the miR-185^high^ groups. In addition, the levels of these four AHAs were not significantly changed at the 3^rd^, 6^th^ and 12^th^ months as compared to the baselines in the follow-up period in both the miR-185^high^ and the miR-185^low^ groups (Fig. 5F–I).

## Discussion

In the present study, we firstly found that the mean levels of plasma miR-185 in DCM patients were significantly higher than those in healthy controls, but there was no correlation between miR-185 and cardiac function at the baselines. Nevertheless, it was unexpected and interesting to note that almost all of these patients could be divided into two different groups according to the cluster distribution of miR-185, which were the miR-185^high^ and the miR-185^low^ groups. During the 1-year follow-up period in patients undergoing the standard treatment for heart failure, the circulating miR-185 expression was stable, but the miR-185^high^ group showed apparent improvements in left ventricular sizes and systolic function, accompanied by the significant decline in the cardiovascular mortality and total admissions for heart failure re-hospitalizations. This indicated that higher the circulating miR-185 levels, the better the clinical outcomes of DCM patients.

Before evaluating the predictive value of miR-185 in DCM development, we had compared the clinical data between miR-185^high^ and miR-185^low^ patients at baseline, and the only difference was the anti-β1-AR antibody content of the blood. Previous studies have demonstrated that ANT, MHC and CC are all intracellular antigens or macromolecules. These molecules depend on CD8-MHC (major histocompatibility complex) class I pathway for antigen presenting in immune response^18^,^19^,^20^. However, only β1 adrenergic receptor depends on CD4-MHC class II pathway for antigen presenting in immune response^21^. Moreover, increasing miR-185 levels attenuate CD4 T cell percentages^22^. Therefore we speculated that miR-185 could down-regulate anti-β1-AR antibody production by inhibiting CD4 T cell development, which influenced MHC class II antigen presentation pathway in DCM patients. In addition, the function of anti-β1-AR antibody is known to disturb cardiomyocyte energy metabolism and promote cell necrosis and apoptosis, and the levels of this autoantibody were continuously repressed in the miR-185^high^ group throughout the entire study, which suggested that miR-185 might take part in alleviating the myocardial injuries mediated by the anti-β1-AR antibody.

MicroRNAs exert their biological effects by regulating the specific target protein expressions^5^. In recent years, miR-185 was proved mainly to inhibit the expressions of relevant immuno-regulatory molecules including Bruton tyrosine kinase and EphB2 in B cells, both of which were associated with B cell activation^11^,^23^,^24^. Besides AHA production, to further clarify whether miR-185 had a potential role in DCM progression through regulating B cells, we detected B cell function in secreting TNF-α, which contributed to low contractility by inducing nitric oxide synthesis, and promoting myocardial fibrosis by facilitating cardiac fibroblasts proliferation and regulating collagen production through ERK1/2 signaling in DCM^4^,^25^,^26^. The changes of TNF-α secreted from B cells were finally observed to be consistent with anti-β1-AR antibody from miR-185^high^ and miR-185^low^ patients. So it could be deduced that miR-185 might be a negative regulator of B cells in DCM, and this inhibitory effect could lead to the improvements of cardiac remodeling and clinical outcomes of DCM patients. In addition to the above, a few experimental studies recently reported that miR-185 was also expressed in hearts^8^, and it could attenuate cardiac fibrosis by down-regulating DNA methyltransferase 1 (DNMT1) activity^27^,^28^,^29^. And Kim, J. O. *et al.* revealed that miR-185 could inhibit cardiac hypertrophy signaling by combining with Camk2d, Ncx1, Nfatc3 and RhoA^30^,^31^,^32^. Furthermore, Xu *et al.* showed that miR-185 could inhibit apoptosis of myocardiocyte by targeting Smad7^33^,^34^. These findings might provide further evidence to strengthen the prognostic value of the miR-185 in DCM. And the potential mechanism is under investigation.

Our study firstly showed that the high miR-185 was associated with the favorable prognosis of DCM patients. The relationship between miR-185 and B cell function implied that gene regulation and immune responses were both important factors for the pathogenesis of DCM. MiR-185 could be a novel biomarker for the clinical outcomes of DCM patients. Targeting miR-185 might be a valuable therapeutic approach for DCM. In addition, the sample size and the follow-up duration of this study need to be improved. And we will expand the sample size to over one hundred DCM patients and prolong the duration of follow-up to two years in the further investigation based on the current finding.

## Additional Information

**How to cite this article**: Yu, M. *et al.* Circulating miR-185 might be a novel biomarker for clinical outcome in patients with dilated cardiomyopathy. *Sci. Rep.* **6**, 33580; doi: 10.1038/srep33580 (2016).

## Figures and Tables

**Table 1:** The Basic Clinical Features of Healthy Controls and DCM Patients

	Healthy controls (n = 41)	DCM (n = 50)	P-value
Age	42 ± 0.31	45 ± 0.28	0.311
Sex (male/female)	25/16	31/19	0.920
Virology (+)			
Coxsachie-B3	4.87% (2/41)	44.00% (22/50)	<0.001
Coxsachie-B5	2.43% (1/41)	28.00% (14/50)	0.001
Cytomegalovirus	2.43% (1/41)	26.00% (13/50)	0.002
Enteric virus	24.40% (10/41)	74.00% (37/50)	<0.001
CRP (mg/L)	0.62 ± 0.02	6.52 ± 0.16	<0.001
NYHA classification			
I	—	0.00% (0/50)	
II	—	4.00% (2/50)	
III	—	64.00% (32/50)	
IV	—	32.00% (16/50)	
LVEF (%)	67.31 ± 1.26	32.57 ± 1.30	<0.001
LVEDD (cm)	4.47 ± 0.17	6.94 ± 0.11	<0.001
NT-ProBNP (pg/ml)	154.27 ± 9.74	3215.02 ± 54.85	<0.001
Medications			
ACEI/ARBs	—	92.00% (46/50)	
β-blockers	—	86.00% (43/50)	
Spironolactone	—	96.00% (48/50)	
Diuretics	—	54.00% (27/50)	
Digitalis	—	52.00% (26/50)	
Nitrates	—	26.00% (13/50)	

Data are presented as the mean ± SEM.

DCM, dilated cardiomyopathy; CRP, C-reactive protein; NYHA, New York Heart Association functional class; LVEF, left ventricular ejection fraction; LVEDD, left ventricular end-diastolic diameter; NT-proBNP, N-terminal pro brain natriuretic peptide; ACEI/ARBs, angiotensin-converting-enzyme inhibitors/angiotensin receptor blockers.

**Table 2:** The Basic Clinical Features of DCM Patients in miR-185^high^ and miR-185^low^ groups.

	miR-185^high^ (n = 22)	miR-185^low^ (n = 24)	P-value
Age	43 ± 0.64	46 ± 0.61	0.531
Sex (male/female)	12/10	13/11	0.979
Virology ( + )			
Coxsachie-B3	45.45% (10/22)	45.83% (11/24)	0.979
Coxsachie-B5	36.36% (8/22)	25.00% (6/24)	0.403
Cytomegalovirus	27.27% (6/22)	25.00% (6/24)	0.861
Enteric virus	68.18% (15/22)	79.17% (19/24)	0.397
AHA (OD)			
anti-ANT antibody	0.309 ± 0.036	0.331 ± 0.039	0.126
anti-β1-AR antibody	0.354 ± 0.020	0.450 ± 0.019	0.022
anti-MHC antibody	0.348 ± 0.031	0.362 ± 0.028	0.095
anti-CC-Abs	0.310 ± 0.025	0.356 ± 0.025	0.086
CRP (mg/L)	4.75 ± 0.313	7.31 ± 0.310	<0.001
NYHA classification			
I	0.00% (0/22)	0.00% (0/24)	-
II	0.00% (0/22)	4.17% (1/24)	1.000
III	63.64% (14/22)	77.67% (16/24)	0.829
IV	36.36% (8/22)	29.17% (7/24)	0.603
LVEF (%)	31.18 ± 1.99	33.37 ± 2.11	0.456
LVEDD (cm)	6.89 ± 0.16	6.95 ± 0.19	0.812
NT-proBNP (pg/ml)	3268.33 ± 124.27	3166.79 ± 108.37	0.898
Medications			
ACEI/ARBs	90.91% (20/22)	95.83% (23/24)	0.499
β-blockers	90.91% (20/22)	83.33% (20/24)	0.446
spironolactone	63.64% (14/22)	70.83% (17/24)	0.603
Diuretics	50.00% (11/22)	54.17% (13/24)	0.777
Digitalis	63.64% (14/22)	50.00% (12/24)	0.351
Nitrates	22.73% (5/22)	16.67% (4/24)	0.605

Data are presented as the mean ± SEM.

DCM, dilated cardiomyopathy; AHA, anti-heart antibody; CRP, C-reactive protein; NYHA, New York Heart Association functional class; LVEF, left ventricular ejection fraction; LVEDD, left ventricular end-diastolic diameter; NT-proBNP, N-terminal pro brain natriuretic peptide; ACEI/ARBs, angiotensin-converting-enzyme inhibitors/angiotensin receptor blockers.

**Table 3:** Re-hospitalization of DCM Patients.

Follow-up time(years)		miR-185^high^	miR-185^low^	P value
1	No. of patients	22	24	
	Total follow-up years	22	23.33	
	Re-hospitalization			
	Patients with ≥1 admission (No. /%)	6 (27.3%)	10 (41.7%)	
	Total admissions (No. /%)	8 (36.4%)	17 (72.9%)	0.011

Chi-square tests were performed for re-hospitalization.

DCM, dilated cardiomyopathy; No., number.
